# Use of machine learning algorithms to classify binary protein sequences as highly-designable or poorly-designable

**DOI:** 10.1186/1471-2105-9-487

**Published:** 2008-11-18

**Authors:** Myron Peto, Andrzej Kloczkowski, Vasant Honavar, Robert L Jernigan

**Affiliations:** 1 Laurence H Baker Center for Bioinformatics and Biological Statistics, 112 Office and Lab Bldg, Iowa State University, Ames, IA 50011-3020, USA; 2 Department of Biochemistry, Biophysics and Molecular Biology, Iowa State University, Ames, IA 50011-3020, USA; 3 Department of Computer Science, Iowa State University, Ames, IA 50011, USA

## Abstract

**Background:**

By using a standard Support Vector Machine (SVM) with a Sequential Minimal Optimization (SMO) method of training, Naïve Bayes and other machine learning algorithms we are able to distinguish between two classes of protein sequences: those folding to highly-designable conformations, or those folding to poorly- or non-designable conformations.

**Results:**

First, we generate all possible compact lattice conformations for the specified shape (a hexagon or a triangle) on the 2D triangular lattice. Then we generate all possible binary hydrophobic/polar (H/P) sequences and by using a specified energy function, thread them through all of these compact conformations. If for a given sequence the lowest energy is obtained for a particular lattice conformation we assume that this sequence folds to that conformation. Highly-designable conformations have many H/P sequences folding to them, while poorly-designable conformations have few or no H/P sequences. We classify sequences as folding to either highly – or poorly-designable conformations. We have randomly selected subsets of the sequences belonging to highly-designable and poorly-designable conformations and used them to train several different standard machine learning algorithms.

**Conclusion:**

By using these machine learning algorithms with ten-fold cross-validation we are able to classify the two classes of sequences with high accuracy – in some cases exceeding 95%.

## Background

Elucidating the relationship between protein sequence and protein structure remains one of the most challenging unsolved problems in computational structural biology. One closely related specific problem is protein designability, that is, why real are proteins not random sequences of amino acids but rather exhibit regular patterns that encode protein structures within the limited number of folds. Reduced (coarse-grained) models of proteins enjoy considerable interest and applicability for studies in designability. In coarse-grained models of proteins a detailed atomistic description of the structure is replaced by a much simpler view where each amino acid is represented by a single point. Additionally, theoretical models of proteins frequently replace the 20-letter amino acid alphabet with a reduced alphabet, up to the limit of a much simpler binary hydrophobic/polar (H/P) representation and furthermore significantly restrict the conformational space by imposing lattices restrictions on the continuous space [1-23]. Through the use of complete enumerations of H/P sequences and compact lattice conformations it has been found that most protein sequences fold to a relatively small number of so called "highly-designable" conformations, while the remaining conformations have few, or no, sequences that fold to them [24-33]. In the present work we use a standard H/P alphabet and a 2D triangular lattice and apply machine learning algorithms to study protein designability for such a reduced model.

Much of the past work on protein designability has focused on searching for the most significant features of designable protein structures, for both lattice models and for real proteins, and relating them to energetic stability and evolution. Recently, it has been shown that proteins selected for thermal stability tend to be more highly designable, owing to their increased energetic stability [34-37]. There is contrary evidence suggesting that designable proteins are unfolded more easily, due to their greater flexibility [38]. Various studies have shown that designable conformations embedded on various lattices exhibit important traits of real proteins, such as symmetrical shapes and secondary structure elements [24-33]. In addition, recent studies suggest that designable lattice structures tend to have more peptide bonds between the protein core and its surface, which can increase protein flexibility [17,38].

Those significant traits of designable conformations, found in previous works, suggested the use of machine learning algorithms to discriminate between sequences folding to highly- and poorly-designable structures. Symmetrical shapes, secondary structure elements, and extraordinary surface-core bonds can possibly appear as definitive patterns in the protein sequences; it has been our intention to exploit such features in this study to classify sequences folding to conformations of differing designability.

In past studies of protein designability amino acid sequences were threaded onto all possible compact conformations for a given shape, and for each case the total energy of the structure was computed based on a specified energy function. If, for a given amino acid sequence, there is a conformation having a total energy lower than all other conformations, it was assumed that the sequence would fold to that specific structure. If many different sequences fold to the same conformation it was assumed that such a structure has high *designablility*. There were also conformations with few or even no sequences folding to them, *i.e*. having poor designability. Additionally many sequences do not fold uniquely, having similar lowest energies for different structures. We may however expect that such a degeneracy effect would rapidly diminish if a simple 2-letter (H/P) amino acid alphabet were replaced with a more complex one. Previous studies that examined the property of protein designability were mostly focused on the conformations within regular lattice shapes in 2D and 3D, such as a 6 × 6 square or a 3 × 3 × 3 cube. Results of these studies imply the existence of only a few highly designable conformations among a much larger number of less or non-designable structures. The results obtained for lattice proteins also suggest that, as for real proteins, designable conformations tend to exhibit structural symmetries. These findings show that a simple lattice model can demonstrate important traits that are mirrored in real proteins.

**Figure 1:**
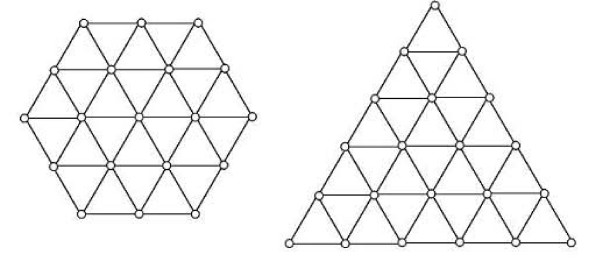
**The hexagonal and the triangular shapes used for the designability studies.** There are 20,843 different compact conformations unrelated by shape symmetries for this hexagon and 22,104 for this triangle.

Our aim is to extend designability studies to various shapes on the 2D triangular lattice and classify sequences folding to highly and poorly designable conformations using machine learning algorithms. The two shapes that are studied here are the triangle and the hexagon, shown in Figure 1. The triangular lattice with the shape of the regular hexagon in Figure 1 has 19 nodes, while the equilateral triangle contains 21 nodes. Therefore there are 2^19 ^(≅ 5.2 × 10^5^) and 2^21 ^(≅ 2.1 × 10^6^) different H/P sequences for each shape. (Our model has no sequence symmetry because of the difference between the C and the N terminals). Because of relatively small numbers of possible H/P sequences and the numbers of all possible compact (no voids allowed) self-avoiding walks unrelated by shape symmetries for the hexagon (20,843) and the triangle (22,104), we are able to enumerate them completely and perform complete designability computations. Similarly, as in previous studies, we find that certain distinct conformations have many sequences folding to those structures, while other have few or no sequences folding to them.

After finding highly- and poorly-designable structures we then compare the sequences that fold to these two classes of conformations and test whether we could classify them by using standard machine learning algorithms. We used the Waikato Environment for Knowledge Analysis (WEKA) software [39,40] available at  as a platform for our classification computations, testing several different algorithms such as Support Vector Machine [41], Naïve Bayes [42] and a Decision Tree [43]. We first trained those statistical learning algorithms on a randomly chosen subset of our data (training set) and then checked the prediction accuracy on a test set. We have performed ten-fold cross-validation experiments to eliminate possible biases. By using a Support Vector Machine with a Sequential Minimal Optimization method of training we are able to obtain highly accurate predictions, often with an accuracy exceeding 90%, depending upon how the binary sequence was represented to the learning algorithm. We are quite optimistic that our approach can also be successfully applied to real proteins to distinguish protein-like sequences folding to distinct native structures from random and non-protein-like sequences that carry no significant structural signal.

## Methods

The complete enumeration of all possible compact conformations for each shape was performed by using a backtracking algorithm generating walks on a tree that checks for all accessible nodes for the next step of the walk. If none of the nodes is available then the algorithm backtracks to the first node offering a different path. Each of nodes must be visited once and only once, with unoccupied voids and chain overlaps not allowed. For longer chains this algorithm suffers from significant attrition and is less efficient than the alternative attrition-free transfer matrix approach developed by us previously [13-15]. However for the relatively short chains containing 19 or 21 nodes studied here a backtracking algorithm is simpler to use. The energy functions that we use for calculating the total energy of each fold obtained by threading of a sequence through a conformation are based only on non-bonded nearest-neighbor contacts. Two neighbors can either be both hydrophobic (with interaction energy E_HH_), one hydrophobic and one polar (E_HP _= E_PH_), or both polar (E_PP_). We use a standard energy function, used in references [24,38], that sets E_HH _= -2.3, E_HP _= E_PH _= -1.0 and E_PP _= 0 in dimensionless energy units. The energy function was derived from real-protein interaction data of amino acids, based on the frequency of non-bonded contacts in protein structures, summarized in the Miyazawa-Jernigan matrix of contact potentials. This function satisfies two significant physical requirements: (i) E_HH _< E_HP _< E_PP _and (ii) 2E_HP _> E_PP _+ E_HH_. The first requirement minimizes the number hydrophobic residues on protein surface, and the second condition allows for the segregation of different amino acid types. This potential will preferentially yield overall a hydrophobic core and a polar exterior.

Because we were interested in a complete enumeration of both the sequence space and the conformational space of our model, we restricted ourselves to the HP binary alphabet. A complete enumeration of the sequence space using the full alphabet of 20 amino acids would not be computationally feasible, as the size of space grows as 20^n^, where *n *is the length of the protein chain. We would need to sample of the sequence space that gives us less insight than the full enumeration. Previous studies have shown, however that the reduced 2-letter HP alphabet model reflects most of the aspects of real protein folding. However, it remains to be seen whether improvements in classification between sequences folding to poorly- and highly-designable conformations could be achieved by using an expanded amino acid alphabet.

In order to classify the sequences folding into highly- and poorly-designable structures we use the WEKA machine learning workbench [39,40] and several classification algorithms, including Support Vector Machine (SVM), Decision Tree, and Naïve Bayes. As input to the statistical learning algorithms we use two different representations of the binary amino acid sequence. Because all sequences for a given shape have the same length (21 residues for the triangle and 19 for the hexagon) it is possible simply to use the binary sequence itself as input. The input vector is thus ***x ***= (*x*_1_, *x*_2_,..., *x*_*n*_) with elements *x*_*i *_(1 ≤ *i *≤ *n*) defined as members of the set *x *∈ {0,1}, corresponding to either a hydrophobic or polar amino acid. In addition, we also tried using as input a percentage count of different tripeptides from the set {HHH, HHP, HPH, PHH, PPH, PHP, HPP, PPP}. The input vector is then ***x ***= (*x*_1_, *x*_2_, *x*_3_, *x*_4_, *x*_5_, *x*_6_, *x*_7_, *x*_8_) with *x*_*i *_(1 ≤ *i *≤ 8) corresponding to the percentage of each *i*^th ^tripeptide in the sequence. Encoding a sequence in this manner allows us to compare sequences of unequal lengths. The resulting classifiers classify a target sequence as either folding to a conformation of high designability or of low designability.

The performance of our classifiers is tested using ten-fold cross-validation experiments, where the data is randomly divided into ten sets, the classifier is trained on nine of the parts, and then the classifier blindly attempts to classify the remaining (known) part. The whole procedure has been repeated ten times using each of the ten sets as a test selection and the final results are compiled. The performance of a classifier can be summarized with the following metrics: *False Positives (FP) *constitute the sequences that fold to conformations of low designability but are incorrectly labeled as folding to conformations of high designability, *True Positives (TP) *are sequences that are correctly labeled as folding to conformations of high designability, *False Negatives (FN) *are sequences that are incorrectly labeled as folding to conformations of low designability, and *True Negatives (TN) *are sequences that are correctly labeled as folding to conformations of low designability. We can define sensitivity and specificity as statistical measures of the performance of the binary classification test, namely:

$$Sensitivity=\frac{TP}{TP+FN}$$

$$Specificity=\frac{TN}{TN+FP}$$

## Results

**Figure 2:**
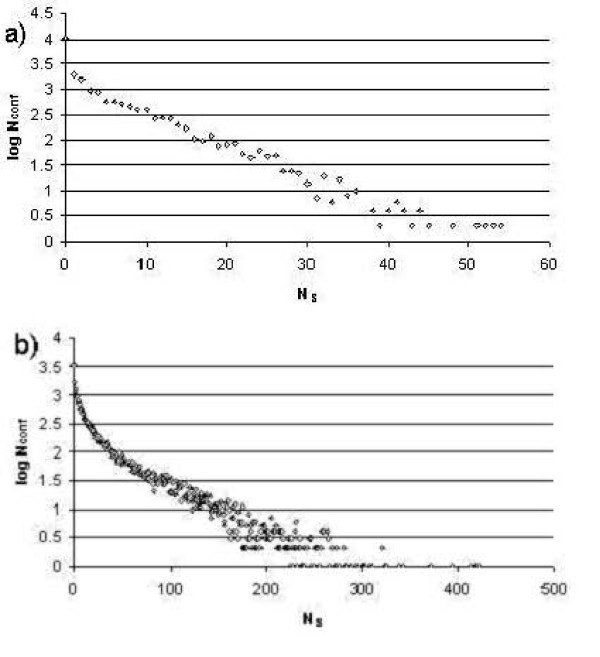
**The dependence of the logarithm of the number of conformations N_conf _on the number N_S of _sequences folding to them. **a) corresponds to data for the hexagonal shape and b) is for the triangular shape.

We enumerate all binary sequences and test them for possible folding to a unique native conformation with the lowest energy among all compact conformations within the given shape. The two shapes studied by us have 19 (hexagon) and 21 (triangle) nodes so the total numbers of sequences are 2^19 ^and 2^21 ^(524,288 and 2,097,152) for the binary H/P case; combined with the 20,843 and 22,104 conformations for each shape, respectively. We then count the number of different sequences folding to a given conformation with energy lower than all other conformations for a given shape and store the counts. These results are shown in Figure 2a for the hexagon, and Figure 2b for the triangle, where the logarithm of the number of conformations log *N*_conf _having *N*_s _sequences folding to them is plotted against *N*_s_. These two graphs express qualitatively the same ideas reported in earlier studies [17,24,28,29,33,38]. There are many conformations with relatively few (or no) sequences folding to them and a rather smaller number of conformations that have many sequences folding to these structures. The latter conformations are named designable conformations and the former are called poorly designable conformations. We used the top 10% and bottom 10% of conformations for the two respective groups.

**Figure 3:**
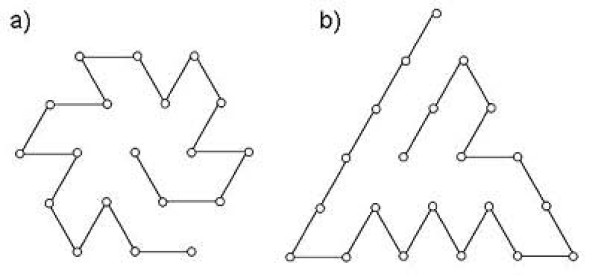
**The most designable conformations for a) the hexagonal and b) the triangular shape.** Conformation a) has 54 sequences folding to it and 11 peptide bonds connecting the protein interior with exterior; conformation b) has 423 sequences folding to it and 9 interior-exterior spanning peptide bonds.

Figure 3 shows the most designable conformations for each shape. The most designable conformation for the hexagonal shape shows features of symmetry that have been found in previous studies [17,24,28,29,33,38]. Both of the conformations contain many peptide bonds between the protein surface and the core, a feature that has been suggested to play an important role in the flexibility of proteins [38].

**Figure 4:**
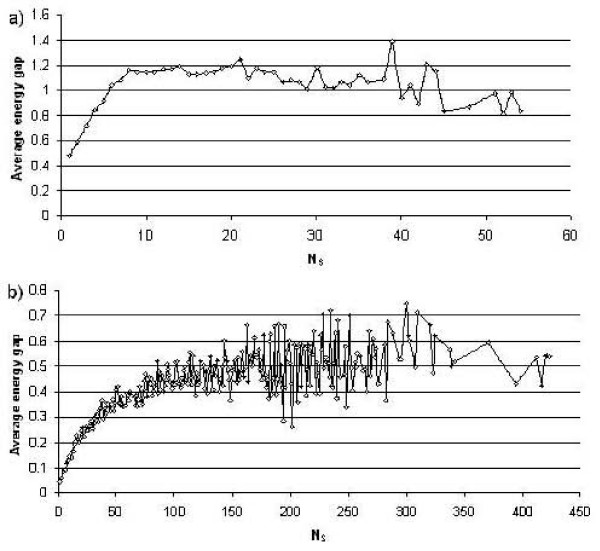
**Average energy difference between the ground state and the next lowest energy state for different values of designability N_S _for the hexagonal (a) and triangular (b) shapes.** Although there is a strong visible trend towards a higher energy gap as the conformations become more designable, there are exceptions particularly for the most designable conformations (corresponding to the largest N_s_), having in both cases average energy gaps below the maximum.

We also show in Figures 4a and 4b the relationship between the average energy gap and the designability of conformations for the two shapes. The energy gap is defined as the difference between the energy of the ground state conformation and second lowest energy conformation for a given sequence. The average energy gap is the average energy gap for sequences folding to conformations of equal designability (N_S_). Similarly as observed in previous studies [24,26,27,38] we find a marked tendency for the energy gap to increase for more designable conformations. This trend seems weaker for larger *N*_s_, which may be a result of having too few conformations to obtain a reliable average. For the hexagonal shape there are fewer than 40 conformations with more than 38 sequences folding to them; whereas there are more than 20,000 conformations with fewer sequences folding to them.

**Figure 5:**
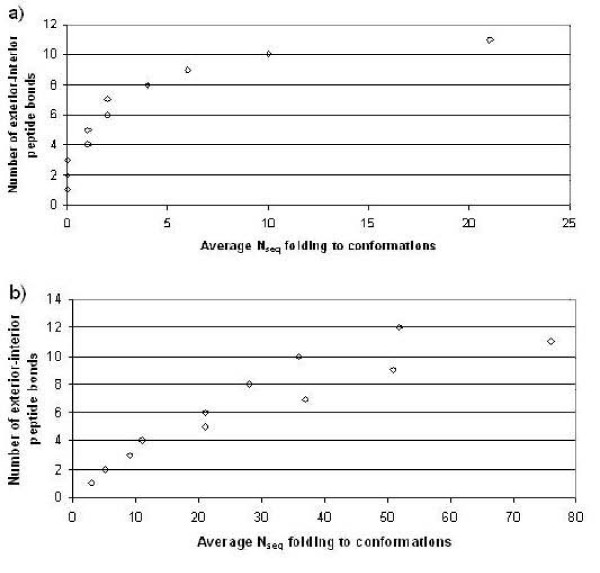
The average number of sequences folding to conformations having the specified number of covalent bonds connecting protein interior with exterior for a) hexagonal and b) triangular shapes.

It has been suggested that the number of peptide bonds spanning between the protein interior and exterior is related to designability, by increasing amount of protein secondary structure and allowing for easier unfolding and folding of the sequence [38]. Previous studies using lattice models have found such a relationship between the number of covalent bonds between the interior and exterior and the protein designability [17,38]. We have computed the average number of sequences folding to conformations having a specified number of peptide bonds between the protein interior and exterior. The results are given in Figure 5 for both the hexagonal (Fig. 5a) and the triangular (Fig. 5b) shapes. Both plots show a strong dependence between the increase in the number of covalent bonds connecting protein interior with exterior and the increase in designability, confirming earlier results in References [17,38].

In addition to the general results presented above, we apply machine learning algorithms to distinguish between sequences folding to highly designable and poorly designable conformations. In our first attempt we define two subsets from the set of all possible sequences: those folding to the bottom 10% of designable conformations and those folding to the top 10% of designable conformations. As there were 54 sequences folding to the most designable conformation for the triangular shape, this would mean, for example, that conformations having 49 sequences folding to them (i.e. within the 10% range from the most designable structure) would be also included in the "highly-designable" set of conformations; and sequences folding to those conformations would be classified as highly-designable sequences. The efficiency of the application of statistical machine learning methods such as SVM depends considerably on the representativeness of the learning sample set used for the training purposes that should include both positive data and the negative ones. We have used both types of these well balanced data for training the models. In order to have a balanced dataset since the number of sequences in both subsets differs greatly, and to reduce the computational cost, we utilize a random sample of sequences from each group. We could not compare sequences corresponding to different shapes, since the triangle has 21 residues while the hexagon has 19.

Table 1 compares the accuracy of prediction obtained by using the J48 Decision Tree, Naïve Bayes, and Support Vector Machine with Sequential Minimal Optimization training. For the J48 classifier, we used a confidence factor 0.25, minimum number of objects 2, and minimum number of folds 3. For the Support Vector Machine classifier we used a c-value 1.0, an epsilon value 1.0E-12, an exponent value 1.0, and a gamma value 0.01. These were the default values provided by the Weka software package. We tried changing these parameters but, aside from differences using different algorithms, we found that varying the parameters did not significantly alter the results. As can be seen from Table 1, we are quite successful in classifying sequences based on whether they fold to highly or poorly designable conformations. All algorithms are consistently above 80% in accuracy, and a Support Vector Machine results in the highest ~95% accuracy. In addition, the area under the curve (AUC), which is a measure of the overall tradeoff between the number of false positives and false negatives, was also high. This indicates that we can achieve a high accuracy with few false positives.

**Table 1:** Accuracy of three different machine learning prediction algorithms – J48 Decision Tree, Naïve Bayes and SVM with SMO training – using binary H/P sequences.^a^

	J48	Naïve Bayes	SMO
a) Sequences folding to the top 10% and the bottom 10% of designable conformations for the hexagon	96.8% correct	95.8% correct	98.3% correct
	AUC .97	AUC 0.99	AUC 0.98
	Sens: 1.0	Sens: 1.0	Sens: 0.997
	Spec: 0.94	Spec: 0.92	Spec: 0.97

b) Sequences folding to the top 10% and the bottom 10% of designable conformations for the triangle	92.7% correct	82.4% correct	95.0% correct
	AUC 0.93	AUC 0.92	AUC 0.95
	Sens: 0.93	Sens: 0.76	Sens: 0.92
	Spec: 0.92	Spec: 0.86	Spec: 0.97

^a ^We compare random subsets of sequences corresponding to the top 10% and the bottom 10% of designable structures for the a) hexagon, and b) triangle. Prediction accuracy and area under the curve (AUC), sensitivity (Sens) and specificity (Spec) for each method are given.

We repeat the above analysis using a different representation of the binary sequences with the sequence being represented by the percent composition of the different tripeptides; for a binary alphabet, there are 8 triplets, HHH, HHP, HPH, PHH, HPP, PHP, PPH, and PPP. Using the frequency of occurrences of such short segments gives us the advantage of being able to compare sequences of varying lengths across different shapes, allowing us to examine whether the designability traits encoded within the binary sequences are a general feature independent of the specific protein shape.

The results in Table 2 show a notable difference in performance for the two shapes (triangle and hexagon), with better results obtained for the hexagon shape over the triangular shape. Neither the Naïve Bayes nor SMO algorithms give indications of the rules that are developed and used to classify sequences as belonging to one group or another. We used approximately 500 poorly-designable and 500 highly-designable sequences for our predictions in tables 1 and 2. We tested a smaller subset of 100 sequences and a larger set of 1500 sequences and found little difference in the accuracies of predictions (results not shown).

**Table 2:** Accuracy of three different machine learning prediction algorithms (J48 Decision Tree, Naïve Bayes and SVM with SMO training) using the frequencies of all possible short tripeptide binary segments.^a^

	J48	Naïve Bayes	SMO
a) Sequences folding to the top 10% and the bottom 10% of designable conformations for the hexagon	89.7% correct	78.8% correct	91.0% correct
	AUC 0.95	AUC 0.92	AUC 0.91
	Sens: 0.91	Sens: 0.85	Sens: 0.84
	Spec: 0.90	Spec: 0.77	Spec: 0.91

b) Sequences folding to the top 10% and the bottom 10% of designable conformations for the triangle	67.8% correct	56.7% correct	57.8% correct
	AUC 0.69	AUC 0.61	AUC 0.58
	Sens: 0.68	Sens: 0.58	Sens: 0.64
	Spec: 0.68	Spec: 0.57	Spec: 0.57

^a ^We compare random subsets of sequences corresponding to the top 10% and the bottom 10% of designabile structures for the a) hexagon, and b) triangle. Prediction accuracy and area under the curve (AUC), sensitivity (Sens) and specificity (Spec) for each method are given.

From the J48 decision tree results we are able to identify the tripeptide sequences containing the most information. For the hexagon shape the two most defining tripeptides are HHH and PPP; for the triangle shape the two most defining tripeptides are PPH and HHH. This means that the percentage of HHH and PPH sequences often was used by the classifier for determining whether sequences were highly- or poorly-designable for conformations in the triangle shape, and likewise PPH and HHP for the hexagon shape. This could be related to the number of interior/exterior peptide bonds, since more interior/exterior bonds would lead to more boundaries between H and P in the triplets (P residues are more often found on the surface and H residues more often in the interior).

**Figure 6:**
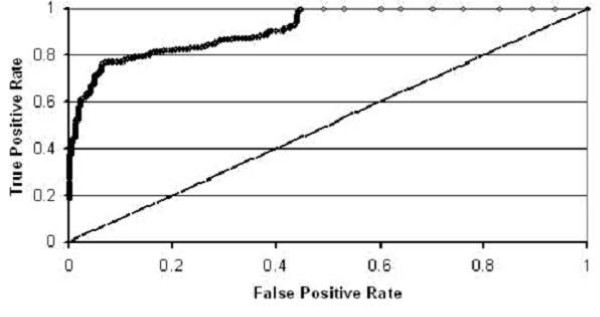
**ROC curve for the Naïve Bayes classifier.** Tripeptide segments are used to classify binary sequences folding to highly- and poorly-designable conformations of the hexagonal shape. The diagonal line y = x, which we would expect if we used a classifier that randomly guessed which class to assign to a sequence, has been added for clarification.

In figure 6 we show a receiver operating characteristic (ROC) curve for the Naïve Bayes classifier on tripeptide sequences in the hexagonal shape. This plot of true sensitivity (true positives found) vs. specificity (few false positives found) gives a visual indication of how our classifier performed. Qualitatively, we see that we obtain a large rate of true positives without having to accept many false positives. This is exactly how we want our classifier to perform and is an indication of the success of the Naïve Bayes classifier on tripeptide segments of sequences folding to conformations in the hexagonal shape.

In order to test whether the ability to distinguish between the two types of sequences might be an artifact, we attempt to classify highly and poorly designable sequences against random binary sequences of the same length. The random sequences are of length 19 for the hexagonal shape and of length 21 for the triangular shape. As previously, we have first randomly sampled sequences from the top 10% and the bottom 10% of designable conformations for the hexagonal and triangular shapes. Then we have randomly sampled sequences of a given length (19 or 21 residues) from the set of 2^19 ^(or 2^21^) possible binary sequences and performed machine learning predictions for all these sets. Tables 3 and 4 show the results for these cases.

**Table 3:** Accuracy of machine learning predictions classifying sequences folding to the most designable conformations among random binary sequences for a) hexagonal and b) triangular shapes.^a^

	J48	Naïve Bayes	SMO
a) Sequences folding to the top 10% of designable structures vs. random binary sequences of length 19 for the hexagon	97.2% correct	94.2% correct	97.3% correct
	AUC 0.97	AUC 0.98	AUC 0.98
	Sens: 1.0	Sens: 1.0	Sens: 0.997
	Spec: 0.94	Spec: 0.89	Spec: 0.95

b) Sequences folding to the top 10% of designable structures vs. random binary sequences of length 21 for the triangle	90.3% correct	84.4% correct	95.2% correct
	AUC 0.91	AUC 0.92	AUC 0.95
	Sens: 0.93	Sens: 0.92	Sens: 0.97
	Spec: 0.90	Spec: 0.82	Spec: 0.94

^a ^Prediction accuracy and area under the curve (AUC), sensitivity (Sens) and specificity (Spec) for each method are given.

**Table 4:** Accuracy of machine learning predictions classifying sequences folding to the least designable conformations among random binary sequences for a) hexagonal and b) triangular shapes.^a^

	J48	Naïve Bayes	SMO
a) Sequences folding to the bottom 10% of designable structures vs. random binary sequences of length 19 for the hexagon	57.5% correct	55.6% correct	57.9% correct
	AUC 0.58	AUC 0.59	AUC 0.58
	Sens: 0.62	Sens: 0.55	Sens: 0.61
	Spec: 0.56	Spec: 0.55	Spec: 0.57

b) Sequences folding to the bottom 10% of designable structures vs. random binary sequences of length 21 for the triangle	50.1% correct	52.3% correct	56.0% correct
	AUC 0.50	AUC 0.53	AUC 0.56
	Sens: 0.54	Sens: 0.67	Sens: 0.59
	Spec: 0.53	Spec: 0.54	Spec: 0.58

^a ^Values of prediction accuracy and area under the curve (AUC), sensitivity (Sens) and specificity (Spec) for each method are given.

For each class there were approximately 300 sequences, chosen to allow a sufficient number to train the classifier but limited by the extent of computations. We test using a larger set of sequences, on the order of 1000, and observe qualitatively the same results as for the smaller set. (The random sequences are generated using standard C++ tools.) In all cases we are careful to ensure that we use two similar sized sets of sequences for our classification tests, as imbalances between the sizes of two classes can artificially enhance the performance of machine learning algorithms.

The general result is that we are quite successful in classifying sequences that fold to highly designable structures among random sequences but are far less successful in classifying sequences folding to poorly- and non-designable structures among randomly chosen sequences. This observation is true of all machine learning algorithms and for both shapes studied.

Finally, in order to further elucidate whether binary sequences carry the shape information in their designability patterns, we attempt to classify both sequences folding to highly designable and poorly designable conformations of the hexagonal shape and the triangular shape. We have also tried machine learning methods to distinguish sequences folding to highly designable conformations folding in the hexagonal shape from poorly-designable sequences folding in the triangular shape as well as highly-designable sequences folding in the triangular shape from poorly-designable sequences folding in the hexagonal shape. Again, because we were classifying binary sequences of unequal lengths, we use the vector of percentages of all tripeptides as the input to our classifiers.

**Figure 7:**
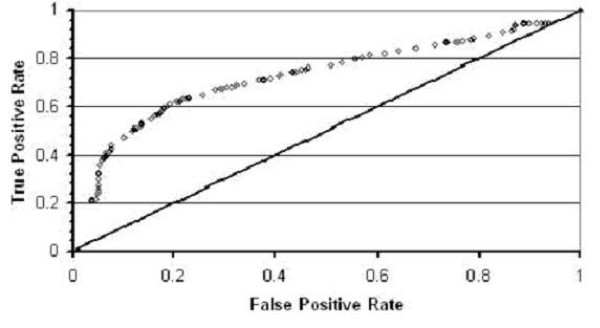
ROC curve for the Decision Tree (J48) classifier. Tripeptide segments are used to classify binary sequences folding to highly- and poorly-designable conformations for both the hexagonal and triangular shapes. The line x = y, expected for the random case is shown for comparison.

In figure 7 we show a receiver operating characteristic (ROC) curve for the decision tree (J48) classifier on tripeptide sequences in both the triangular and hexagonal shape. In this case our classifier performs worse than in the case of single sequences (hexagonal) but is still significantly better than random guesses. This suggests there is some important signal from the tripeptide segments of binary sequences folding to both shapes.

From Table 5 we see that, although there are wide disparities among different classification algorithms and between different shapes, in general we are relatively successful in classifying sequences folding to different shapes based upon the composition of different tripeptides for the sequence representation. It is also surprising how well the Decision Tree algorithm (J48) classifies sequences folding to different shapes, in comparison with the other algorithms. When we more closely examine the tree output from the WEKA software package we found that the tripeptide sequence PPP of three sequential polar residues carries most of the structural information. This means that the percentage of PPP tripeptide segments was a particularly strong indicator of which class (designable vs. non-designable) a sequence would fold to. As mentioned earlier, we speculate that this is related to the number of interior/exterior peptide bonds. Conformations with fewer interior/exterior bonds would have correspondingly more segments of pure H or pure P, thus leading to the result seen.

**Table 5:** Accuracy of machine learning predictions.^a^

	J48	Naïve Bayes	SMO
a) Sequences folding to the top 10% of designable structures vs. sequences folding to the bottom 10% of designable structures for both shapes	69.5% correct	65.0% correct	65.6% correct
	AUC 0.73	AUC 0.69	AUC 0.67
	Sens: 0.67	Sens: 0.66	Sens: 0.71
	Spec: 0.71	Spec: 0.65	Spec: 0.64

b) Sequences folding to the top 10% of designable structures of hexagonal shape vs. sequences folding to the bottom 10% of designable structures in the triangular shape	98.1% correct	84.9% correct	87.0% correct
	AUC 0.99	AUC 0.92	AUC 0.87
	Sens: 0.98	Sens: 0.82	Sens: 0.84
	Spec: 0.98	Spec: 0.90	Spec: 0.92

c) Sequences folding to the top 10% of designable structures of triangular shape vs. sequences folding to the bottom 10% of designable structures in the hexagonal shape	98.0% correct	65.8% correct	64.3% correct
	AUC 0.99	AUC 0.70	AUC 0.63
	Sens: 0.98	Sens: 0.64	Sens: 0.75
	Spec: 0.98	Spec: 0.72	Spec: 0.66

^a ^For classifying a)sequences folding to highly-designable conformations for the hexagonal and triangular shapes against sequences folding to the least designable conformations for these two shapes; b)sequences folding to the most designable conformations of the hexagonal shape against sequences folding to the least designable conformations of the triangular shape and c)sequences folding to the most designable conformations of the triangular shape against sequences folding to the least designable conformations of the hexagonal shape. Prediction accuracy and area under the curve (AUC), sensitivity (Sens) and specificity (Spec) for each method are given.

## Discussion

The protein structural designability results obtained in the present paper for two regular shapes on the 2D triangular lattice are not qualitatively different from results obtained in numerous earlier studies [17,24,28,29,33,38]. We found that designable conformations having many sequences folding to them are relatively rare among a large number of conformations that have few or no sequences folding to them with the lowest energy. We have also found that the average energy gap between the ground state and next lowest energy state increases with increasing designability of structures; similarly as observed earlier by [24,26].

The most interesting results obtained in our present study relate to our ability to successfully classify sequences folding to highly- and poorly-designable conformations using several standard freely available machine learning algorithms. For both of the shapes studied (the hexagon and the triangle) we are able to classify successfully the sequences using their full binary representation, which we may ascribe to the fact that there are relatively few highly designable conformations, and sequences folding to them probably share similar patterns in the distribution of hydrophobic and polar residues along the protein sequence.

That there was a significant difference in the classification accuracy between the two shapes came as a surprise to us. The hexagonal shape, being more compact, resembles real protein structures more than the less compact triangular shape. There were a similar number of total conformations for each shape, even though the triangular shape had 21 vertices and the hexagonal shape had only 19. Perhaps the corners of the triangle placed restrictions on all of the conformations such that the differences between the poorly- and highly-designable conformations were less pronounced. This could lead to smaller differences between the sequences folding to each set and hence poorer classification accuracy.

Additionally, our further testing of sequences folding to the most designable structures among completely random sequences seems to suggest that the structural designability pattern is somehow encoded in the sequence. If the structural designability information is indeed encoded in the binary sequence we would expect to discern sequences folding to highly designable structures among random sequences much more effectively than sequences folding to poorly-designable structures. The results of our computations fully support these expectations. We are able to classify sequences folding to highly-designable structures among random sequences with an accuracy exceeding 90%; whereas for sequences folding to poorly- and non-designable structures our accuracy of prediction among random sequences was not much better than random. Our testing of sequences folding to designable conformations in different shapes suggests that the overall shape of the fold may also be encoded in the protein sequence.

The results presented here lend further support to the use of simple H/P lattice models developed for protein structural studies. Our success in classifying sequences folding to conformations in the triangular lattice, a lattice without the parity effects of the square or cubic lattice, offers evidence of the usefulness of such simple models. As mentioned earlier, an interesting next step would be to test our machine learning algorithms on sequences of real proteins which fold to higher or lower designable states. Recent work [35-37] finds that proteins of thermophilic organisms tend also to be more designable than proteins in mesothermic organisms. We are working on classifying these two sets of protein sequences using the same tools used in this study. It would be rather remarkable if a designability footprint exists for real protein sequences.

The real protein folding problem is, of course, significantly more complicated than folding on simple lattices with a reduced 2-letter HP alphabet. The present success in applying statistical machine learning algorithms to distinguish between highly-designable and poorly-designable sequences for lattice proteins suggest that similar approach can be applied to real proteins. Statistical machine learning algorithms are already extremely useful in bioinformatics for prediction of protein secondary structure from the amino acid sequence, prediction of protein classes, protein-protein, protein-RNA, protein-DNA, or protein-ligand binding sites, prediction of intrinsically disordered regions in proteins, prediction of phosphorylation and other post-translationally modified sites, and many other purposes. The main problem is a proper choice of training (positive and negative) sets for the learning process. It is a difficult endeavor, since sometimes a single mutation changes protein structure. We are currently working on this problem for real proteins and hope that our approach will help to a certain degree in protein folding studies.

## Authors' contributions

MP conceived of the study, carried out most computations. AK conceived of the study, wrote program for enumeration of compact conformations. VH supervised machine learning methodology and statistical analysis of the results. RLJ conceived of the study, and participated in its design and coordination. All authors read and approved the final manuscript.
